# An outbreak of gastroenteritis associated with a novel GII.8 sapovirus variant-transmitted by vomit in Shenzhen, China, 2019

**DOI:** 10.1186/s12879-020-05643-x

**Published:** 2020-12-01

**Authors:** Yuxiao Yan, Yuan Li, Wen Shi, Xiangyu Kong, Huiying Li, Qing Zhang, Lili Pang, Li Jiang, Junying Liu, Miao Jin, Yuning Li, Zhaojun Duan

**Affiliations:** 1 The First School of Clinical Medicine of Lanzhou University, Lanzhou, 730000 Gansu China; 2 Department of Viral Diarrhea, NHC Key Laboratory of Medical Virology and Viral Diseases, National Institute for Viral Disease Control and Prevention, Chinese Center for Disease Control and Prevention, 155 Changbai Rd, Chang-ping District, Beijing, 102206 China; 3 Shenzhen Baoan Center for Disease Control and Prevention, Baoan District, Shenzhen, Guangdong Province China; 4 Yingkou Center for Disease Control and Prevention, Yingkou, Liaoning China; 5 The First Hospital of Lanzhou University, Donggang-xi Rd, Cheng-guan District, Lanzhou, 730000 Gansu China; 6 Central Hospital of Zhoukou, Zhoukou, 466000 Henan China

**Keywords:** Sapoviruses, Outbreak, Vomit, Genome, Phylogenetic analysis

## Abstract

**Background:**

Human Sapoviruses (SaVs) has been reported as one of the causative agents of acute gastroenteritis (AGE) worldwide. An outbreak of SaVs affected 482 primary school students during spring activities from February 24 to March 11, 2019 in Shenzhen City, China. Our study was aimed at determining the epidemiology of the outbreak, investigating its origins, and making a clear identification of the SaVs genetic diversity.

**Methods:**

Epidemiological investigation was conducted for this AGE outbreak. Stool samples were collected for laboratory tests of causative agents. Real-time reverse-transcription polymerase chain reaction (rRT-PCR) and conventional RT-PCR were used for detecting and genotyping of SaVs. The nearly complete genome of GII.8 SaV strains were amplified and sequenced by using several primer sets designed in this study. Phylogenetic analysis was performed to characterize the genome of GII.8 SaV strains.

**Results:**

The single factor analysis showed that the students who were less than 1.5 m away from the vomitus in classroom or playgroundwere susceptible (*P* < 0.05). Seven of 11 fecal samples from patients were positive for GII.8 SaV genotype. In this study, we obtained the genome sequence of a SaV GII.8 strain *Hu/SaV/2019008Shenzhen/2019 /CHN (*SZ08*)* and comprehensively analyzed the genetic diversity. The phylogenetic analysis showed that the GII.8 strain SZ08 formed an independent branch and became a novel variant of GII.8 genotype. Strain SZ08 harbored 11 specific amino acid variations compared with cluster A-D in full-length VP1.

**Conclusions:**

This study identified SaVs as the causative agents for the AGE outbreak. Strain Hu SZ08 was clustered as independent branch and there was no recombination occurred in this strain SZ08. Further, it might become the predominant strain in diarrhea cases in the near future. Constant surveillance is required to monitor the emerging variants which will improve our knowledge of the evolution of SaVs among humans.

## Background

There has been increasing concern about Sapoviruses (SaVs), a member of the family *Caliciviridae*, as a causative agent of gastroenteritis in humans in both sporadic cases and outbreaks worldwide, especially among infants and young children [1–5]. SaVs are recognized as the second most commonly etiological virus behind Norovirus in children with acute diarrhea after the successful deployment of the Rotavirus vaccine [6]. SaVs-associated outbreaks have occured in semi-closed institutions such as kindergardens, schools, hospitals and nursing homes for the elderly, and happen through the fecal-oral route, the primary route of transmission. SaVs transmit via exposure to SaVs-positive aerosols from feces, vomitus or the consumption of SaVs-contaminated food and water [7, 8]. SaVs-associated acute gastroenteritis (AGE) has increased and has been accepted as a major public health problem worldwide, especially in developing countries [6, 9, 10].

The genome of SaVs consists of a single-stranded positive-sense RNA genome of approximately 7.1–7.5 kb in size and has a 3′-end poly (A) tail [7]. The SaVs genome contains either 2 or 3 open reading frames (ORFs). ORF1 encodes nonstructural proteins and a major capsid protein (VP1). ORF2 encodes a minor structural protein (VP2). ORF3 predicted for several SaVs strains encodes proteins with unknown function [2, 7, 9].

Based on complete capsid gene (VP1) sequences, SaVs are classified into at least 15 genogroups (GI − GXV), four of which (GI, GII, GIV and GV) are known to infect humans. The human SaVs in genogroups GI, GII, GIV and GV are currently subdivided into 7 GI (GI.1-GI.7), 8 GII (GII.1-GII.8) and one GII.NA, 1 GIV (GIV.1) and 2 GV (GV.1 and GV.2) genotypes [11], and one additional genotype of GII.NA that has been reported recently [12].

Recently, a novel genotype of SaVs, GII.8 strains were reported in some countries and one GII.8 SaV strain (GZ2014-L231) was detected in 2014 in Guangzhou City, Guangdong province of China [13]. In this study, we reported an AGE caused by a novel GII.8 SaV variant occurred in primary school in Shenzhen City, Guangdong province of China in 2019. The genome of the GII.8 strain was analyzed and the epidemiology of this outbreak was described. The emerging of novel GII.8 SaV variant made a warning for SaVs outbreak control and prevention.

## Methods

### Epidemiological investigation

Cases were defined as at least three bouts of unformed (loose and watery) stool and/or vomiting episodes in a 24-h period during the outbreaks. A standardized questionnaire was prepared to collect demographic data (gender and age), and illness onset data (symptoms and duration of symptom).

### Nucleic acid extraction

A 0.1-g fecal specimens was diluted with 1.0 ml phosphate-buffered saline (pH 7.2) to a 10% suspension. The supernatants were collected and the viral genomes were extracted by using the QIAamp Viral RNA Mini Kit (Qiagen, Hilden, Germany) and the QIAamp DNA Mini Kit (Qiagen), according to the manufacturer’s instructions.

### Bacteria isolation

The stool samples were analyzed for major bacterial pathogens (*Escherichia coli*, *Salmonella*, *Shigella*, *Campylobacter* and *Yersinia enterocolitica*) with MacConkey agar (MAC, Oxoid Ltd., Basingstoke, UK), *Salmonella Shigella* agar (Oxoid Ltd), Campylobacter-selective agar (Oxoid Ltd) and *Yersinia* Selective agar (Schiemann’s CIN agar, Oxoid Ltd), respectively.

### Virus detection

TaqMan real-time reverse-transcription polymerase chain reaction (rRT-PCR) was performed to test all viral nucleic acid for Norovirus and SaVs with primers and probes as described previously [14, 15]. By using enzyme immunoassay Kits and rRT-PCR, Rotavirus A was identified [16]; and Astrovirus was identified using rRT-PCR [17]; rRT-PCR was performed to detect Adenovirus [18]. Conventional RT-PCR was performed using primer sets including P289 and P290 (in polymerase region) and SLV5317 and SLV5749 (in capsid region) to further characterize positive samples for SaVs as described previously [19]. The PCR cycling conditions were as follows: 42 °C for 30 min, 95 °C for 15 min followed by 40 cycles of 95 °C for 1 min, 50 °C for 1 min, and 72 °C for 1 min, and a final 10-min elongation at 72 °C. The temperature was held at 4 °C until use.

### Nearly complete genome amplification

The nearly complete genome of GII.8 strains were amplified by using 14 primer sets (Table 1) which were designed based on the whole genome of the reference strains MG674584 and MF462287. The primer set of SLV5317 and VN_3_T_20_ [19] was used to amplify the complete capsid and ORF2 sequences. PCR products were sequenced using an ABI3730XL DNA Analyzer (Applied Biosystems, Foster City, CA).

**Table 1:** Primers used in this study for sapovirus detection and amplification of almost whole genome

	Primer	Nucleotide sequence (5′-3′)	Position	Source
**Amplification of almost complete genome**	orf-1	GAGTTGAACACGCAGTCC	42-59^a^	**This study**
		ATGATGGCACCAGTAAGG	1009-1026^a^	
	orf-2	CGCAGTGTCAGTGGTGTC	880-897^a^	
		AAGGTATGGTCGAACGAA	1880-1897^a^	
	orf-3	CGTCGTGTTCTAACTGTTGA	1763-1782^a^	
		GAATCTTGTGATAACCTCCATA	2740-2761^a^	
	orf-4	CCAATTAGTAGCTGAAACCCTT	2719-2740^a^	
		CCCTTCCACGCAAACACG	3634-3651^a^	
	orf-5	CAGAGGGCACTTATGAGAC	3432-3450^a^	
		CAGAGGCAGTTATGGGAG	4434-4451^a^	
	orf-6	GGGTCGTGTATTGTTTGG	4335-4352^a^	
		CCGAGTTTGGCATTTCTA	5280-5297^a^	
	orf-7	TAGTGTTTGAAATGGAGGGC	5157-5176^a^	
		TTGGGAAGTGACTGCTGA	6164–6181^a^	
	orf-8	AGGGCATCATCTTTCCAC	6114–6131^a^	
		TTTGCGAGACAGTCCATTA	7029-7047^a^	
	orf-9	GGAATCAACTGCGGAAAT	6577-6594^a^	
		GAATAAGACAGCGATGGTC	7429-7447^a^	
	AP1	CTCGCCACCTATGAAGCA	5081-5098^a^	
		AATCGGCAGTGATGTCCT	7004-7021^a^	
	Orf1–1	CTTCTAAGCCATTCTACTCAA	5-25^b^	
		CGTGCCATGAACTGTTTG	1179-1196^b^	
	Orf1–2	GGCAGCATCACATCAGTC	1093-1110^b^	
		TGTTTGCAGACATGAACG	2078-2095^b^	
	Orf1–1,2	CTTCTAAGCCATTCTACTCAA	5-25^b^	
		GCTCTGTTTGCAGACATG	6206-6323^b^	
	Orf1–4	ACCGTGGGTGGTATGACT	2479-2496^b^	
		GGCGACAACCGTTGAAAT	3269-3286^b^	

^a^Position in the complete sapovirus GII genogroup sequence (accession no: MG674584)

^b^Position in the complete sapovirus GII genogroup sequence (accession no: MF462287)

### Genotype and sequence analysis

These nucleotide sequences were first verified using Geneious 10.1.3 (www.geneious.com). Sequences were assembled by DNA Star software. Phylogenetic analysis of these sequences was performed in the MEGA6.0 software package (http://www.megasoftware.net/). Sequence Alignment was performed using Clustal W. Best substitution models for the dataset were chosen based on the lowest Bayesian Information Criterion score. Phylogenetic trees with bootstrap analysis from 1000 replicates were generated by using the neighbor-joining method. SimPlot software was used to identify potential recombination among known genotypes of SaVs. DNAMAN software was used to compare the nucleotide and the amino acid identity of strain *Hu/SaV/2019008Shenzhen/2019 /CHN* (SZ08) with reference strains.

### Data analysis

Statistical analysis was performed with the IBM SPSS Statistics software (Version 26.0 International Business Machines Corporation, Armonk, New York, United State). Odds ratio (OR) and 95% confidence interval (CI) of categorical variables were calculated using two tailed, and the comparison of categorical variables among different groups are conducted with Chi-square or Fisher’s exact test. Quantitative variable was described as mean, median, standard deviation or inter-quartile range (IQR). Quantitative variable was compared by rank-sum test, analysis of variance or t test. Significant difference was considered as the level of *P* < 0.05 with two-tailed test.

### Nucleotide sequences and accession number

The SaV sequences determined in this study were deposited in GenBank under the accession number MT561022 for the nearly full genome sequences.

## Results

### Epidemiological investigation

**Fig. 1:**
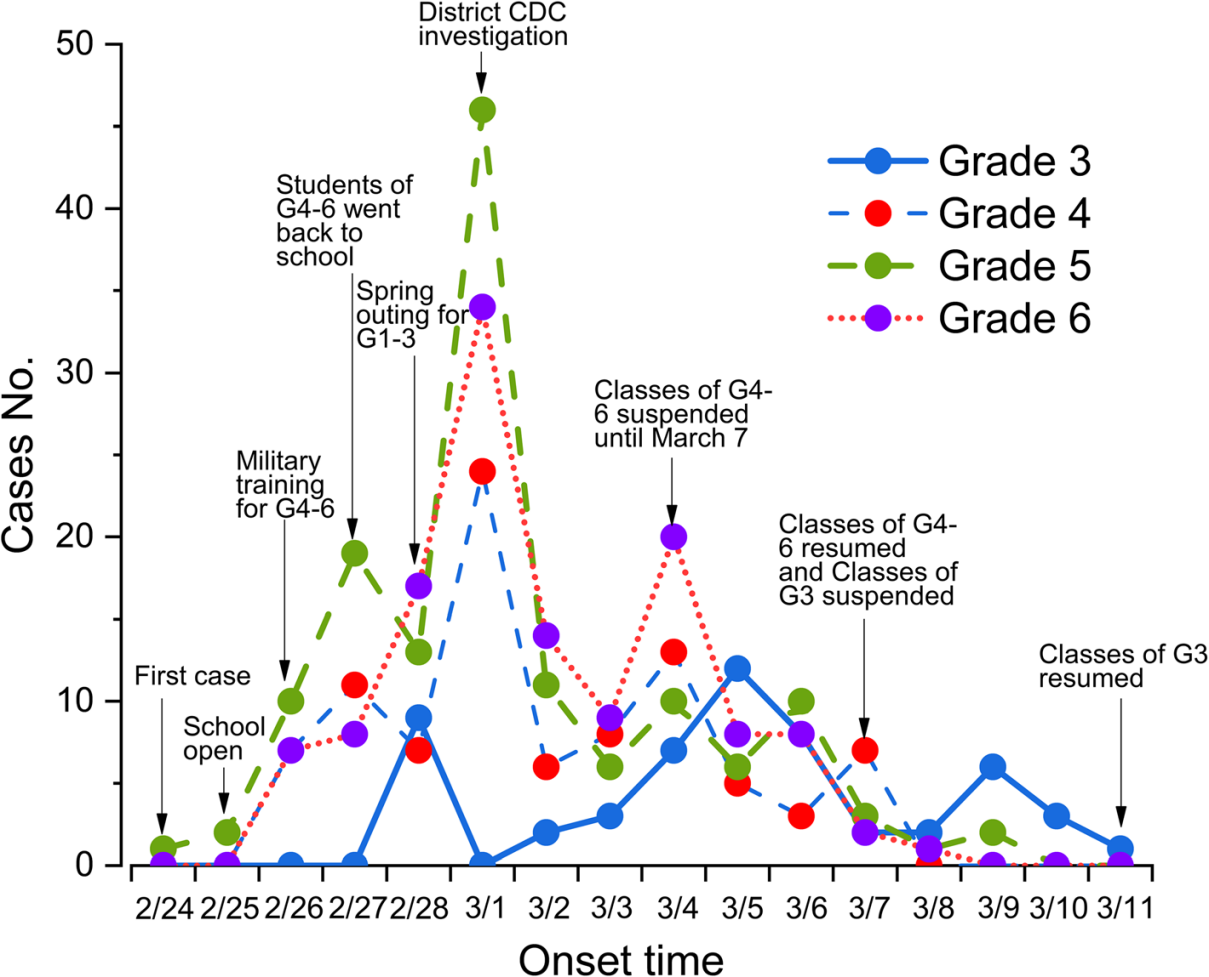
Epidemic curve of acute gastroenteritis cases in the outbreak occurred in Shenzhen, China, 2019

**Fig. 2:**
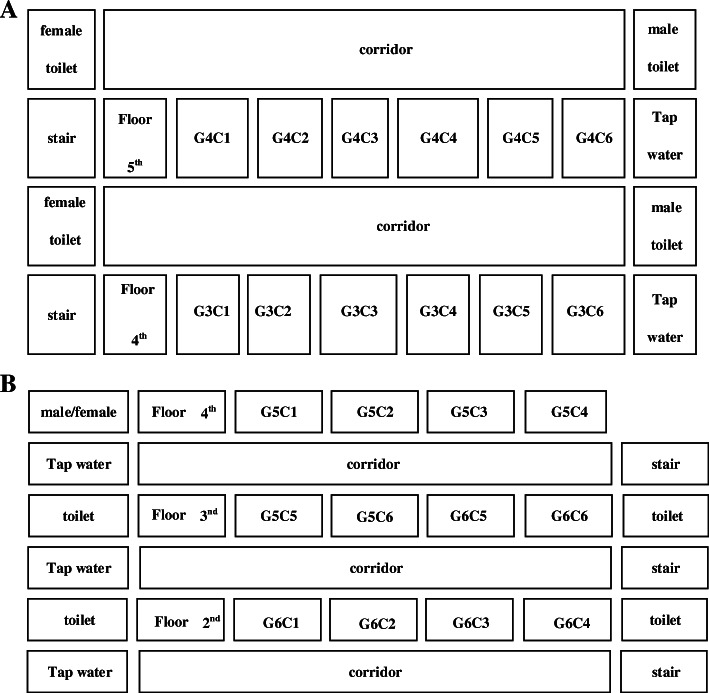
**a** Schematic diagram of each floor of BuildingA. **b** Schematic diagram of each floor of BuildingB

This AGE outbreak occurred in a primary school in Shenzhen City in China. It started on Feb 24th and lasted until March 11th 2019. It included 482 cases among the 1850 students and 95 teachers of the school. The school has 34 classes divided into 6 different grades. There were two buildings in which students of grade 3 and 4 were in Building A and students of grade 5–6 were in Building B. The students from grade 4–6 were organized to participate in military training activities during February 26–27 while the students from grade 1–3 were organized to participate in spring outing on February 27 after the school opened on February 25. The age of 482 cases ranged from 9 to 14 years. The first case came from class 4, grade 5, and had symptoms one day (February 24th) before school began after winter vacation (February 25th). He did not rest at home, still returned to school on school day and vomited in classroom. During the subsequent military training activities, he vomited on the bus (February 26th). The students of grade 5 got sick first and then grade 4, 6 and 3. There was no case from grade 1 and 2. Peak of cases occurred on March 1 on which local district CDC was invited to investigate this outbreak and the second peak of cases occurred on March 4 on which classes of G4–6 were suspended and were resumed on March 8. Classes of G3 were suspended on March 8 and resumed on March 11 (Fig. 1). The characteristic symptoms within the cohort were diarrhea (67%, 321/482), vomiting (45%, 218/482), dizziness (30%, 144/482), stomach pain (22%, 106/482) and nausea (18%, 86/482). The results showed that the attack rate of each grade was 36% in Grade 5, 23% in Grade 6, 21% in grade 4 and 6% in grade 3, respectively (*P* < 0.05). The attack rate of case each floor of Building A was 3% for the 4th floor, 20% for the 5th floor (x^2^ = 45.09, *P* < 0.01), and the attack rate of case each floor of Building B was 41% for the 3rd floor, 38% for the 4th floor, and 20% for the 2th floor (x^2^ = 16.44, *P* < 0.05). The difference of case incidence among different floors was statistically significant (Fig. 2a, b).

During the epidemic, 218 students vomited. Vomiting occurred in plastic bags (13%), floor (10%) and window (1%) while riding the bus and vomiting occurred in the bathroom (3%) and dormitory (2%) during activity. Vomiting occurred in Classrooms (9%), playgrounds (3%), hallway (3%) after returning to school (Table 2). In the group with common exposure to vomit, the single factor analysis showed that among the students that saw other peers vomiting in the classroom or playground, being at a distance of 1.5 m or less of the vomiting event which provided more chances to access to the virus, make them more susceptible to the infection (*P* < 0.05) (Table 3).

**Table 2:** Distribution of vomiting places of students

Place of vomiting	No. of vomiting cases	%
Bus		
Plastic bag	28	13
Ground	10	5
Outside the window	2	1
Military training		
Bathroom	6	3
Dorm	4	2
Playground	4	2
Canteen	1	0
School		
Toilet	61	28
Classroom	20	9
Playground	7	3
Doorway	6	3
Other	13	6

**Table 3:** Case control study on risk factors of acute gastroenteritis outbreak caused by Sapovirus

No.	Case		Non case		OR value	95%CI		X^2^	*P* value
	Expose	%	Expose	%		lower limit	Upper limit		
See others vomiting	278	58	365	46	1.58	1.26	1.99	15.6	0.00
See others vomiting in the classroom	120	25	138	17	1.56	1.19	2.06	10.14	0.00
See others vomiting in the playground	36	7	35	4	1.74	1.08	2.81	5.22	0.02
Less than 1.5 m from vomit	156	32	166	21	1.42	1.04	1.95	4.81	0.03
Handling vomitus	45	9	36	5	2.17	1.37	3.41	11.57	0.00
See others Vomiting on bus	86	18	116	15	1.25	0.92	1.7	2.11	0.15
See others Vomiting during activity	14	3	30	4	0.76	0.4	1.44	0.72	0.40
See others Vomiting in bathroom	56	12	71	9	1.33	0.92	1.93	2.28	0.13
Self protection in handling	12	27	7	19	1.93	0.69	5.4	1.62	0.20
Family members in the same school	116	24	170	22	1.15	0.88	1.51	1.06	0.30

### Pathogen investigation

A total of these pathogens, including *E. coli* Salmonella, Shigella, Campylobacter, Yersinia enterocolitica, Norovirus, Rotavirus, Adenovirus and Astrovirus were detected to be negative. Sequence analysis of several positive rRT-PCR samples revealed identical sequences, which were identified as SaVs. SaVs were detected in 7 of 11 stool specimens collected from the cases of the outbreak .

### Analysis of nearly complete genome sequence

The nearly complete genomic sequence of strain SZ08 was obtained and its length was 7338 nt excluding the sequences of 5’UTRs and the 3′-end poly (A) tail in this study. The sequenced genomes were predicted to contain two major ORFs from 1 to 6837 (ORF1, encoding the nonstructural proteins and major capsid protein VP1) and from 6838 to 7337 (ORF2, encoding the minor structural protein). Its 3′-UTR had 52 nt. The reference strain MF462288/ GII.8/Peru330/PNV010961/2008 shared the highest query cover and nucleotide identities (93.8%) with the strain SZ08 based on nearly complete genome. The nucleotide identities of the strain SZ08 with strain Peru330 were 93.13% in the ORF1, 94.61% in ORF2 and 93.30% in ORF3, respectively. According to the amino acid identities of the protein alignment, a great degree of similarity (96.95% at the nearly complete genome, 98.46% in the nonstructural proteins and VP1 and 97.54% in the VP2) were shown between them. We observed 48 aa variations in both nonstructural proteins and VP1 and 25 aa variations in the minor structural protein.

### Analysis of complete capsid protein

**Fig. 3:**
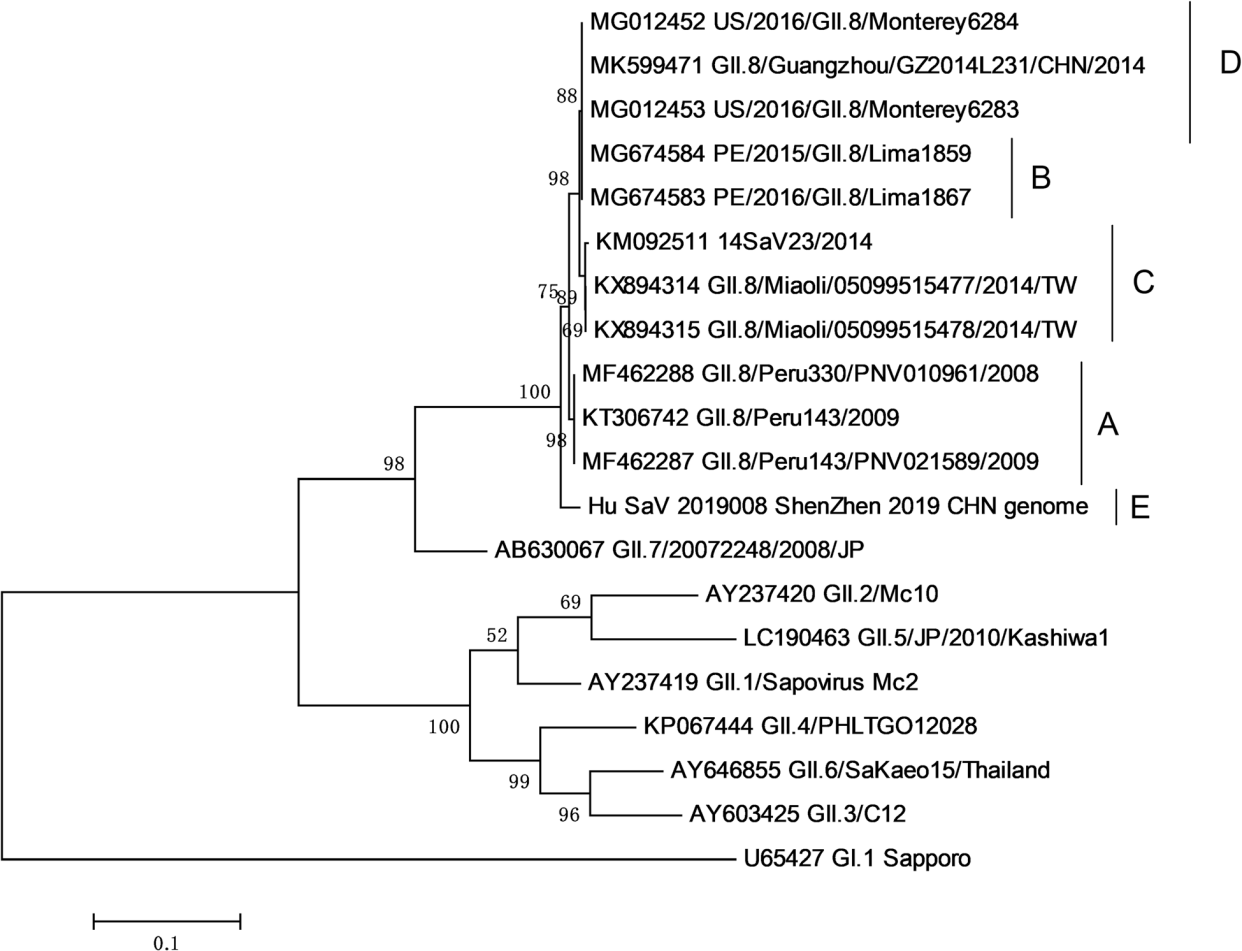
Phylogenetic analysis of SaVs based on nucleotide of compete VP1

**Fig. 4:**
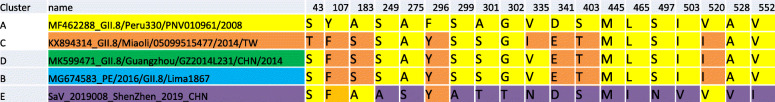
Amino acid variation in complete capsid protein gene compared to reference strains of SaV GII.8

According to the phylogenetic analysis, Strain SZ08 clustered into GII.8 branch and was independent with other clusters (A, B, C and D) named previously [13] and was named after cluster E (Fig. 3). The nucleotide identity of the strain SZ08 was ranged from 92.94 to 94.02% with cluster A-D and the amino acid identity was ranged from 96.76 to 97.48%. However, cluster A-D shared the high nucleotide identity (96.87–99.84%) and amino acid identity (98.31–99.88%). Strain SZ08 harbored 11 specific amino acid variations (S249A, A275S, S299A, A301T, G302T, V335N, L465I, S497N, I503V, A528V and V552I) compared with cluster A-D. However, five original amino acids had the transversion (S43T, S183A, E341D, T403S, I520V) from the earliest strain Peru330/PNV010961/2008 (cluster A). Cluster B-D had low amino acid variations (Fig. 4).

## Discussion

The epidemiology of cases of AGE of viral etiology is a relevant public health issue [20]. Of note, reports on SaVs-associated AGE across all age groups have recently increased worldwide [6, 13, 21], which indicated that the cases caused by SaVs may become extensive and global. And Shenzhen is also one of the high-incidence areas of SaVs-associated outbreaks recently in mainland China [19, 22]. Human SaVs cause AGE in all age groups in both sporadic cases and outbreaks worldwide as well as predominantly occurs in children [23, 24], which correspond to this study. On the other hand, the outbreak described by the study occured in primary school and expose to vomit was recognized as the cause, which are similar to the previous studies [25, 26].

The investigation showed the difficulty to prove the person-to-person transmission by contact or from aerosols generated by vomit [27–29]. In this study, we investigated the vomiting places and the single factor analysis showed that the participation in the handling of vomitus and the distance of vomitus were the main factors causing gastroenteritis infection (logistic regression, *P* < 0.05). It has been proposed that the formation of aerosols that can remain in the air for some time and can then be breathed in and swallowed or through contamination of surface causes the outbreaks [30–32]. The ingestion of aerosolized vomitus, aerosols transmission via vomiting might explain the partial gastroenteritis outbreaks which are not caused by foodborne or waterborne outbreaks as described previously [32–35]. Vomit was considered only when exposure was at < 1 m, however, possible exposures at greater distances have also been reported [27, 28]. Our study showed the distance under 1.5 m could cause the infection. Therefore, the study gave the evidence that the greater distance also could cause the cases. Since vomiting is a common symptom in calicivirus outbreaks, ingestion of aerosolized vomitus, may exist in most SaVs outbreaks. Therefore, ingestion of aerosolized vomitus, should be valued in the investigation of gastroenteritis outbreaks [28].

Emerging virus strains often have a risk of causing a pandemic. SaV GII.8 was first identified in two hospitalized children’s samples in Peru, in 2008 [36]. In mainland China, GII.8 was first reported in Shenzhen, in 2011 [22]. Additionally, in 2019, Xue al. acquired the first GII.8 SaV genome from mainland China [13]. Based on the phylogenetic analysis, the strain SZ08 in this study was classified as a member of GII.8 SaV. In addition, strain SZ08 isolated in 2019 was clustered as independent branch and was different from the strain GZ2014-L231 isolated in 2014 although strain of Shenzhen and strain GZ2014-L231 of Guangzhou were both from the same province, Guangdong. GII.8 strains were also detected in other countries not only in clinical but also in environmental samples [37, 38]. SaVs outbreak caused by GII.8 strains occurred in long term center in previous report [36] and the GII.8 SaV outbreak occurred in primary school. It suggested the GII.8 strain could cause the children and the elderly to infect. Despite the low detection rate, the wide spread distribution of the virus in different countries and wide age groups, make it as important concern to understand its genetic diversity and evolutionary characteristics.

Recombination and mutations are the two important way of viral evolution not only in SaVs but also in Noroviruses [13, 39]. According to the genomic analysis, there was no recombination occurred in strain SZ08.

Amino acid variation in protein is the important way of viral evolution. In this study, by comparing the differences between the strain SZ08 as new cluster and GII.8 human SaV representative strains of other clusters, more amino acid variation occurred in VP1 capsid region in which potential epitopes and receptor binding sites located. The variation in VP1 might be one of the explanations why there was the large scale of cases in this SaVs outbreak in this study.

In conclusion, contacting vomitus could be one of important transmission mechanisms which may explain a large number of cases of SaVs outbreaks. It is noteworthy that SaVs-associated diarrhea is generally mild, while large-scale outbreaks by SaV may also occur. In addition, we obtained the genome of a novel GII.8 SaV variant from China and comprehensively analyzed the genomic characteristics. The results of this study could not only provide reference data for SaVs researches in the future, but also deepen the understanding of evolution mechanisms of the new GII.8 variant. Constant surveillance is required to monitor the emergence of these strains and will make a clear identification of changes in major strains and improve our knowledge of the evolution of SaVs among humans.

## Data Availability

The datasets used and analyzed during this study are available from the corresponding author on reasonable request.

## Abbreviations

SaVs: Sapoviruses
AGE: Acute gastroenteritis
rRT-PCR: Reverse-transcription polymerase chain reaction
ORFs: Open reading frames
OR: Odds ratio
CI: Confidence interval
IQR: Inter-quartile range
